# Robust inference of the context specific structure and temporal dynamics of gene regulatory network

**DOI:** 10.1186/1471-2164-11-S3-S11

**Published:** 2010-12-01

**Authors:** Jia Meng, Mingzhu Lu, Yidong Chen, Shou-Jiang Gao, Yufei Huang

**Affiliations:** 1 Department of ECE, University of Texas at San Antonio, San Antonio, TX, USA; 2 Department of Pediatrics, University of Texas Health Science Center at San Antonio, San Antonio, TX, USA; 3 Greehey Children's Cancer Research Institute, University of Texas Health Science Center at San Antonio, San Antonio, TX, USA; 4 Department of Epidemiology and Biostatistics, University of Texas Health Science Center at San Antonio, San Antonio, TX, USA

## Abstract

**Background:**

Response of cells to changing endogenous or exogenous conditions is governed by intricate molecular interactions, or regulatory networks. To lead to appropriate responses, regulatory network should be 1) context-specific, i.e., its constituents and topology depend on the phonotypical and experimental context including tissue types and cell conditions, such as damage, stress, macroenvironments of cell, etc. and 2) time varying, i.e., network elements and their regulatory roles change actively over time to control the endogenous cell states e.g. different stages in a cell cycle.

**Results:**

A novel network model PathRNet and a reconstruction approach PATTERN are proposed for reconstructing the context specific time varying regulatory networks by integrating microarray gene expression profiles and existing knowledge of pathways and transcription factors. The nodes of the PathRNet are Transcription Factors (TFs) and pathways, and edges represent the regulation between pathways and TFs. The reconstructed PathRNet for Kaposi's sarcoma-associated herpesvirus infection of human endothelial cells reveals the complicated dynamics of the underlying regulatory mechanisms that govern this intricate process. All the related materials including source code are available at http://compgenomics.utsa.edu/tvnet.html.

**Conclusions:**

The proposed PathRNet provides a system level landscape of the dynamics of gene regulatory circuitry. The inference approach PATTERN enables robust reconstruction of the temporal dynamics of pathway-centric regulatory networks. The proposed approach for the first time provides a dynamic perspective of pathway, TF regulations, and their interaction related to specific endogenous and exogenous conditions.

## Background

Gene and protein regulatory networks depict the regulatory circuitry that controls the complicated biological processes. The regulatory networks enable visualization and identification of protein and gene functions that cannot otherwise be revealed by a gene- or protein-centric approach. Through revealing various system level interactions including transcription regulation, signal transductions, and metabolic reactions, regulatory networks provide insights into the molecular machinery of cell. The systems perspective also reveals global regulatory properties of cell including functional modularity and robustness to perturbation. Regulatory networks are also at the heart of personalized medicine, where they serve to aid disease diagnosis and prognosis, drug target discovery, intervention, and prediction of pharmacological effects [1].

Reconstructing regulatory networks comprises one of the most active areas of research in computational biology. The goal of network construction is to elucidate the gene/protein interaction relationships, in an effort to obtain more realistic and predictive models of molecular regulation. This prompts us to study the complex patterns of regulatory network that depend on temporal and condition-specific context.

### Regulatory networks are specific to exogenous context

Since gene or protein in the network can acquire different functions under different exogenous conditions or phenotypic contexts, the network constituents and interactions would be context dependent, i.e., regulatory network is composed of different genes and proteins under different conditions and their interactions are specifically determined by the context. For instance, the oncogenes such as MYC, ERK, and WNT are known to down-regulated in normal cells. Up-regulation of oncogenes leads to a series of modification in signal transduction and transcription regulation, which interfere with the controlled cell death and eventually turn the normal cell into cancer cell. Therefore, the revelation of context-specific differences of regulatory network in cancer is essential for understanding the process and mechanism of cancer. In fact, the context-dependent regulation accounts for the heterogeneity of diverse cell phenotypes.

### Regulatory networks are time varying according to the endogenous cell states

While the long run behaviour of regulatory networks is often stationary, the transient characteristic is intrinsically time varying for they need to respond and adapt themselves to the new exogenous condition. Temporal dynamics of regulatory networks is highly prevalent to many important processes such as cell cycle, cell differentiation, apoptosis, and viral infection to account for different endogenous cellular states. Even under a fixed exogenous condition, the regulation of genes and protein are carried out in a temporal, sequential manner. The cell cycle is known to have multiple stages and transitions in between and are triggered and guided by temporal changes of regulatory networks. Some of the transcription factors such as Fos are known to be early genes because they regulate commonly at the early stage of a biological event. It is also well recognized that the regulatory pathway does not always persist over all times. Such temporal cascade of regulation is essential for elucidating the mechanism leading to different phenotypes. The two key characteristics of time-varying networks include 1) the temporal topology of regulatory networks, i.e., the network constituents and wiring could change with time, and 2) time-varying regulatory impact. It is important and necessary to understand both aspects of temporal regulation when reconstructing the time varying networks.

### Existing network models and approaches are insufficient for inferring context-specific time-varying networks.

Reconstruction of regulatory networks based on high throughput data is currently a research focus in systems biology. In the past, a large number of different network architectures have been proposed. One of the most popular architectures is the molecular-based network architecture, where nodes are individual genes or proteins and links denote gene-gene, gene-protein, or protein-gene regulations. This architecture aims to reveal the detailed genomic interaction and is capable of uncovering transcription regulation, signalling, and metabolic pathways. However, the quality of the inferred molecular-based network is greatly restricted by the limitation of data availability and data quality; in practice, precise inference of large scale networks at a molecular level is very difficult and not robust due to the large number of the unknowns and limited and noisy genomics or proteomics data. The reconstructed networks normally have extremely high false positive links, making the subsequent analysis and interpretation at a molecular level difficult and unreliable. A common remedy to this problem is to take a global view of the networks and study the features such as scale free and modularity. Although this approach has been successful in revealing global properties of molecular networks, it is still unable to identify pathways as intended by this architecture. An alternative and equally popular architecture is the module networks [2]. Since nodes in a module network are clusters of genes, or modules, the inference task for module networks is less demanding and higher quality inference can be achieved. However, module networks cannot directly uncover functional pathways even with high quality data and thus are often difficult to interpret biologically. Post-processing based on gene ontology (GO) or pathway enrichment is required to reveal the possible functions of each modules. However, the module networks cannot reveal beyond the prediction that a set of functions of a modular would regulate another set in another module. This prediction is useful for pointing out the possible further studies to follow; it is nevertheless far from sufficient to uncover important pathways and transcription regulations. Apparently, a new architecture that is robust to data noise and capable of revealing directly pathways will be much more appealing.

Given a network structure, a mathematical or statistical model needs to be imposed to model the dynamics of regulation. Many models have been proposed for this purpose including ordinary differential equations [3], (probabilistic) Boolean networks [4], [5], information theory based models [6]. However, the principled assumption underlying these models is overwhelmingly stationary; that is the dynamic models do not change over time. While the long run behaviour of regulatory networks is reasonably considered stationary, the transient characteristic is intrinsically time varying for they need to respond and adapt themselves to meet the need of varying conditions. As a result, the inferred networks based on stationary assumption conceal the intricate temporal regulatory patterns. Despite the prevalent temporal nature of regulatory, limited work exists to model and infer the time varying network and they include regime state space model [7], temporal hidden Markov model [8], the wavelet dynamic vector autoregressive method [9], and context-sensitive probabilistic Boolean networks [10]. These models all involve a high degree of parametric complexity that hinders robust reconstruction of large scale networks. For instance, context-sensitive probabilistic Boolean networks in [10] and regime state space model [7] involved only 10 and 47 genes, respectively. Alternatively, module networks were assumed in [7-9] to increase the inference robustness but nevertheless reduce the interpretability of result. Some remarkable results about systems level reconstruction of both static and transient transcription regulatory networks of yeast for different cell states were reported in [11] .The networks inference utilized a large collection of prior knowledge and a simple model of differential expression in microarray. However, specific time regimes for cell states of interest need to be given; the approach cannot further uncover by itself endogenous temporal patterns within the given regime. In addition, the adopted gene networks architecture is sensitive to data noise and prone to producing false positive links.

Aside from modelling network dynamics, investigation of the context-specific networks is given increasing attention, although the main body of existing work still concerns the reconstruction of context-independent networks. To this end, [12] explicitly introduced a context node in the Bayesian network model to model different context condition. GO annotations were applied in [13] to the protein-protein interaction (PPI) network of yeast to dissect the context (function)-specific subÂ¬networks. Again, in [11], large scale transcription networks under different exogenous conditions were constructed for yeast. Despite some success in identifying context-specific network motifs and transcription factors, the results were not robust and unreliable at the molecular scale due to the molecular-based network architecture..

To sum up, making robust statistical inference from diverse types of genomic data to discover context-specific and temporal pathways of biological function presents a formidable challenge. The current network architectures and approaches are insufficient for this goal. The main issue is that the network architecture, model, and inference approach cannot produce robust network inference that can be easily understood.

### Towards a pathway-centric network model and functional enrichment inference approach

To overcome the problems of existing network architecture, model, and inference approaches, we advocate a new pathway-centric network (PathRNet) and an enrichment inference approach. The nodes of the PathRNet are regulators such as transcription factors (TFs) ,pathways including signaling pathways and metabolic pathways, and the links bear specific functional meaning, i.e., a link going from TF to pathway defines the transcription regulation of pathway by TF and a link from signaling pathway to TF suggests signal transduction induced modification of TF. The advantages of this model architecture are in four folds. First, this network design is geared directly to biologists, who normally focus on specific pathways and regulators when designing experiments and seeking answers to phenotypic outcomes. Secondly, PathRNet speaks the language of biologists and no interpretation is even needed for the results. Unlike in most of gene or protein networks, where links only imply a degree of association and hence further interpretable is needed, in PathRNet, the linkage such as Signaling pathway A -> TF B -| Metabolic pathway C reads naturally as Signaling pathway A activates TF B, which further inhibits metabolic pathway C. Thirdly, past research has produced considerable data about pathways and transcription regulation, and it is prudent and necessary to understand their regulatory roles in different context before new pathways and regulations can be inferred. Fourthly, PathRNet is more robust to data noise. Since nodes are pathways instead of individual genes, PathRNet inherits the robustness of module networks; given these advantages, we believe that PathRNet can adequately address the aforementioned deficiency of the existing network architectures.

To robustly infer the context specific structure and its temporal dynamics of PathRNet, we propose the Pathway Enrichment Analysis of Temporal Regulatory Networks or PATTERN. The key of PATTERN is the method of the enrichment based time dependent clustering algorithm, ECTDISA [14], which identifies temporal clusters of pathways and regulated gene sets that are enriched in a context-specific time series microarray data. Once again, the inference in PATTERN moves away from the gene-centric approach to the pathway or gene-set enrichment based concept. The gene set enrichment (GSE) concept formulated by the GSEA algorithm [15] has gained great success in differential analysis of gene expression data and also extended its applications to drug effect prediction and clustering of coregulated gene. The main advantages of GSE approach are 1) the inference is robust to excessive noise on one or a few genes in the gene set, and 2) the result has straightforward biologically implications since gene sets are mostly pathways or coregulated genes. Recalling the previously mentioned problems of current inference approaches for network reconstruction, we see that these advantages of GSE are the exact answers to these problems. However, GSE has not been used for network construction. It is thus our objective to develop an enrichment approach for network reconstruction. Coupled with PathRNet, the GSE approach will provide added robustness to inference, enabling reliable discovery of network temporal dynamics from noisy microarray data of commonly small sample size.

## Methods

### Overview of our approach

**Figure 1:**
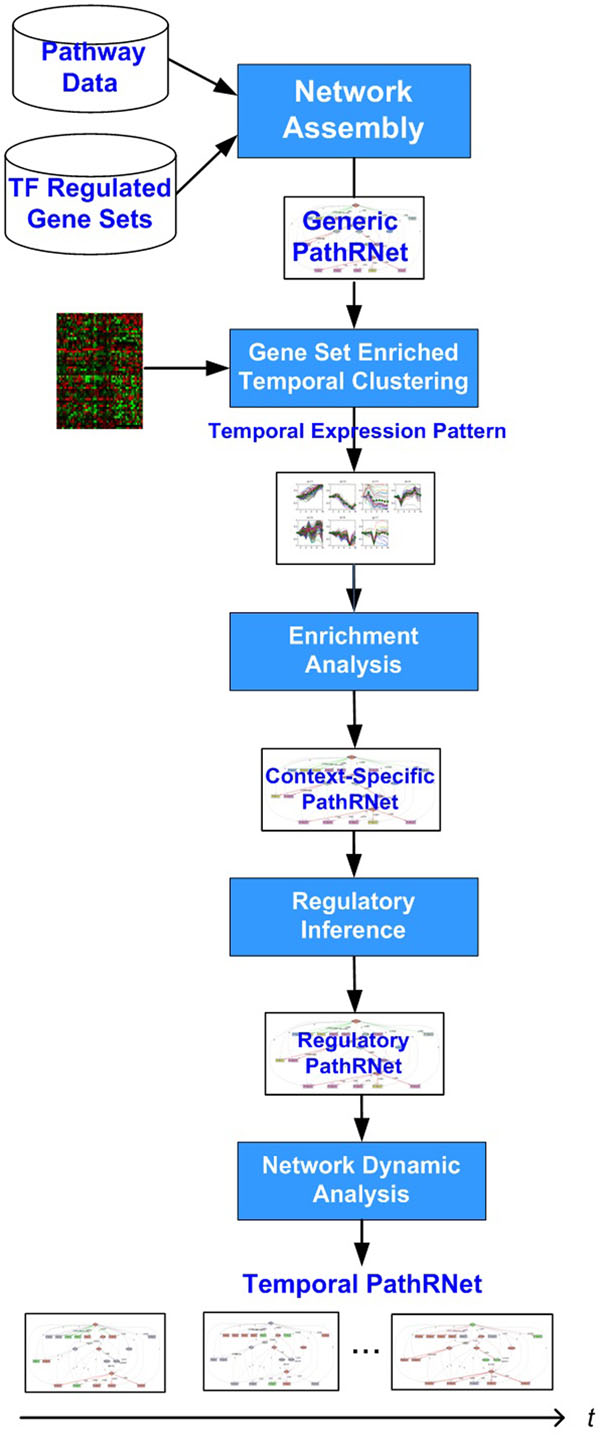
**Block diagram of PathRNet Algorithm** The construction of PathRNet follows a modular structure, namely generic PathRNet construction, context-specific PathRNet construction, regulatory PathRNet construction, and temporal PathRNet construction.

The block diagram of our approach PATTERN is shown in Fig. 1. It has a modular structure including four modules. In module 1, pathway data and TF regulated gene sets data from the existing knowledge database are assembled to construct a generic PathRNet. To construct the network, only two types of directed links are allowed, i.e., a link from a signaling pathway node to a TF node is placed if TF is in the downstream of the signaling pathway and a link from a TF node to a signaling or a metabolic pathway is placed if the TF regulated gene set contains genes in the pathway. The resulting PathRNet is a generic network that represents the prior knowledge of regulation under variety of different contexts. In Module 2, context-specific microarray data is introduced and the context-specific PathRNet is obtained by a functional enrichment strategy. Particularly, ECTDISA [14] is applied to determine the enrichment of each node in the generic PathRNet in the time series microarray data and the nodes that are not enriched will be deactivated. Furthermore, ECTDISA also determines the temporal expression pattern (the expression trend and time span) of each node. In Module 3, regulatory PathRNet is constructed to depict the regulatory relationship of nodes based on the expression pattern obtained in Module 2. Inference is carried out to determine the presence of links and their regulatory impact. As a result, a (context-specific) regulatory PathRNet will be obtained. In Module 4, temporal regulatory impact of network during each time period is further analyzed, resulting in a temporal landscape of PathRNet dynamics.

We discuss next the proposed methods for implementing each module.

### Generic PathRNet Construction

A generic PathRNet is first constructed based on the existing knowledge database. The prior knowledge databases are collected from NCI-NC [16] ,Transfac [17] and MSigDB [15]. A generic PathRNet is constructed with each node corresponding to a signaling pathway, a transcription factor or a metabolic pathway; and an edge is placed:

a) From a signaling pathway to TFs if TFs are the downstream of the signaling pathways regulate transcription factors.

b) From a TF to signaling or metabolic pathways if TF regulated at least one genes in the pathway.

In the end, a prior network is obtained, which illustrates a generic picture of the interactions between pathways and transcription factors inside a human cell.

Noted that in our approach, there are additional steps to refine the constructed generic PathRNet by removing redundant nodes and edges:

In the context-specific PathRNet construction module, all the nonfunctional nodes and their edges were removed, leaving only the enriched functional pathways /TFs and edges between them.

In the regulatory PathRNet construction module, Bayesian information criterion (BIC) was applied for model selection, and all the edges that are not chosen were removed from the constructed regulatory PathRNet.

In the dynamic PathRNet construction module, (dynamic) edges that indicate no or nominal contributions were further removed from the constructed temporal PathRNet.

Since all the following steps remove redundant nodes/edges, we hope keep as many associations as possible when constructing the generic PathRNet to ensure a high sensitivity of reconstruction. Thus, an edge is placed between a transcription factor (TF) and a pathway as long as the TF regulates at least one gene in the Pathway (the pathway and the TF target gene sets share at least one gene). However, the degree of overlapping between the pathway genes and the TF target gene set might itself indicates the strength of their association. Although this information is not used in our algorithm, it is possible to include this information in further extension to better estimate the regulatory impacts of transcription factors.

### Context specific PathRNet construction

To capture the functional components of generic PathRNet and construct a context-specific PathRNet, expression profile is then incorporated. The goal is to identify coregulated pathways enriched by the gene expression profile and their associated time period. To this end, we apply the ECTDISA [14] to the microarray gene expression data. ECTDISA can not only cluster the coregulated genes that are functionally most enriched by a functional annotation database such as pathway databases but also retrieve the timing of the co-regulating clusters.

Particularly, when applying the ECTDISA, a measurement needs to be specified to gauge the coregulation of genes. Since Pearson's correlation is not robust towards noise, and Euclidean distance is not suitable to measure correlation, we proposed a modified Euclidean distance measurement *ρ* to penalize smaller fold changes without losing the robustness to noise, which is defined as follows:

where *x*, *y* are the expression profile of a functional node (pathway or TF), and *α* is used to penalize smaller fold changes.

**Figure 2:**
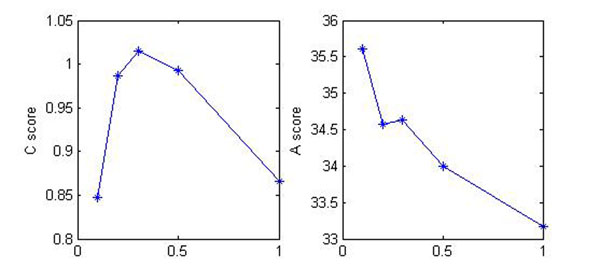
**Choose appropriate value for *α*** Different values of *α* were used when PATTERN was applied to real data, and the results are compared in terms of A score and C score [18]. We chose *α* to achieve the highest C score.

Since small fold changes are more vulnerable to noise, the parameter *α* was chosen to penalize smaller fold change to a reasonable amount. It should not be too small or too large. An experiment is developed on real data to show the sensitivity of the parameter, and result is evaluated in terms of *A* score and *C* score, which are defined in [18] for performance evaluation purpose. In general, *A* score indicates whether all the identified modules are corresponding to true modules; *C* score indicates whether all the true modules are correctly identified by clustering algorithm. Since our goal is to choose a proper *α* to uncover all the modules from the data, we are mainly interested in acquiring the highest *C* score; but at the same time, *A* score should not be too low. The result is shown in Fig. 2.

It can be seen from the figures that *C* score reaches its peak when *α* = 0.3 and at the same time *A* score is relatively high. This is the value we choose when the approach was applied to real data. To achieve the best result, the use of different *α* are recommended.

**Figure 3:**
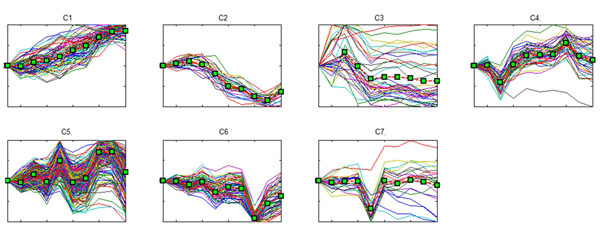
**Modules identified by ECTDISA** ECTDISA can uncover both temporal transcriptional modules (C3) and constant time modules (C1, C2, etc). Starting from 189 initial gene sets, ECTDISA was able to identify 119 clusters in the KSHV infection data, which were further merged into 7 modules. The 3rd module (C3) is a very good example of a temporal module, where genes of the module behave quite differently between 0-3 hours but share a common trend afterwards.

An example of clusters produced by ECTDISA is shown in Fig. 3. A number of the most significantly enriched signaling pathways, transcription factors and metabolic pathways are selected as the functional components in the prior network, and the context specific PathRNet is constructed by retaining only the functional components.

### Regulatory PathRNet construction

Note that for now the edges in the functional networks are still defined by the prior knowledge. With the expression data, the edges can be further refined to reflect the regulatory status including up- or down- regulation and its weight. To this end, the expression profiles of the nodes are first determined. For the real case, since even the genes in the same pathways can behave differently, i.e., some are up regulated while some others are down regulated, it possible that the same pathway are enriched in both the up-regulated module and the down-regulated module. To capture the main trend of pathway, we consider the most significantly enriched trend in a pathway by using ECTDISA algorithm. Specifically, for a signaling pathway or a metabolic pathway (nodes labelled with "S-" and "P-" in the network), the expression profile is estimated by averaging all the pathway genes that are most enriched in their temporal trends. Since the trends of their expression profiles are very similar, the averaging expression of those genes is a good estimate of the main trend of the pathway. For example, if at 1 hour, the expressions of 5 genes that are involved in the same pathways are 2, 2.1, 2.2, 0, -3; and the first 3 gene are enriched in a module identified by ECTDISA, then the fold change of the corresponding pathway at 1 hour will be . Similarly, the expression fold change at other time points can be calculated. Note that, since the expression is calculated by averaging along the gene dimension, the expression fold change of different genes are acquired from exactly the same experimental condition; moreover, the microarray data has been properly normalized [19]

If a pathway is enriched in a temporal transcription module, then the pathway is only active during the inferred time period that the temporal module is active; otherwise its expression is 0. For a transcription factor, the expression fold change profile is inferred by averaging the expression fold changes of its encoding genes.

Next, the regulatory impact is inferred based on the expression of nodes. Let *y* denote the expression profiles of a node and **X** = [*x*_1_*,**x*_2_,...,*x**_k_*] the expression profiles of its parent nodes. Then, a linear regulatory model is introduced as:

*y** = ***X***·**β** + **ε*

where *β* represents regulatory weights and *ε* is white Gaussian noise. The goal is then to determine the functional parent nodes and the corresponding regulatory weights *β*. The problem is a variable selection problem and BIC is adopted for the solution. Under the assumption that the model error is normally distributed, the formula for BIC is:

where *n* is the number of data points in *x**_i_*, *k* is the number of parameters to be estimated, and the residual sum of squares (RSS) is the sum of squares of residuals, which defines the discrepancy between the data and our estimation model:

RSS=|| *y**-*ŷ||^2^

where,  is an estimate of *y* calculated based on the least squares estimate:

### Temporal PathRNet construction

In the last module, the dynamic regulatory network is constructed from the basal network by considering the dynamic contribution of the regulatory nodes at each sample time points. Specifically, the dynamic change of the expression level of a node at a sample time *t* can be expressed as:

where, *x**_t_* = [*x*_1,_*_t_*, *x*_2,_*_t_*,…,*x_p,t_*], and *P* is the number of parent nodes of *y*. The dynamic contribution of all the parent nodes is (*x**_t_* – *x**_t_*_–1_)âˆ™*β*, and the dynamic contribution of a parent node from *t* – 1 to *t* is: . Then, the dynamic regulatory networks can be obtained by refining the edges at each *t*. Particularly, if  is small, the corresponding edge can be removed indicating no or nominal contribution. Otherwise, the width and the color of the edge will be defined by  to illustrate the regulatory influence of the node onto a child node during a particular sample time period. Although small regulatory contribution  does lead to edge removal and thus change of network structure, it is mainly the regulatory time period inferred by the ECTDISA that defines the dynamics of the network structure.

## Simulation results

**Figure 4:**
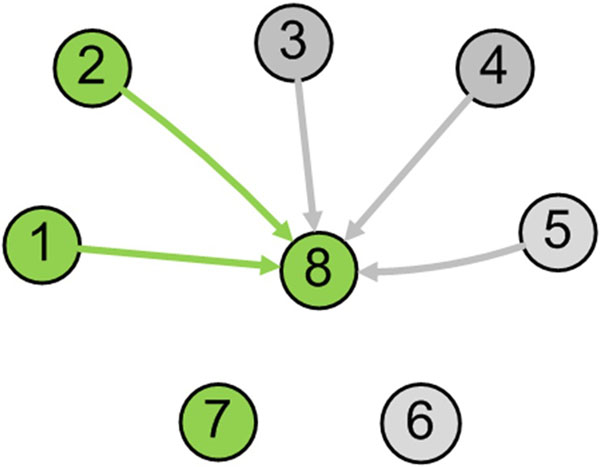
**Simulated PathRNet** The simulated PathRNet consists of 8 nodes and 5 edges in the generic network, which corresponds to 8 pathways or TFs and 5 regulations. Node 8 is assumed to be regulated by node 1-5 according to prior knowledge but only regulated by node 1 and 2 in the context specific PathRNet. Node 7 is another functional node but does not regulate node 8.

PathRNet was first validated on simulated data. The data was constructed to mimic a microarray experiment that measures the expression profiles of 200 genes at 9 sample time points. 8 temporal transcription modules were assumed to exist in the expression data, each of which contains 30 to 50 genes and may share the same subset of genes. Genes in a module was assumed to be co-expressed and follow a coherent expression pattern [20]. A simple PathRNet (Fig. 4) was devised with each node corresponding to a molecule. Moreover, only nodes 1, 2, and 8 were assumed functional in the simulated expression data. The function of each node and their constitute genes were defined in a simulated prior knowledge database; the database contains a set of assumed pathways and TF regulated gene sets, whose members may be the genes in the module. Specifically, in the prior knowledge database, node 8 was assumed to be regulated by node 1-5. Note that only nodes 1 and 2 are functional nodes in the context-specific PathRNet that underlies the simulated expression data.

The data pattern of each module was generated according to an AR (1) model:

*X_mt_ =* 0.9*X_m_*_(_*_t_*_–1)_*+ε_mt_*, where, *X_mt_* represents the expression level of module *m* at time *t*, and *ε*_mt_ ~ Normal(0,1^2^). Then, the expression level of gene *g* in module *m* time *t,* can be simulated as:

where *G_m_* denotes the set of genes in module *m* and *T_m_* is the duration of module *m*.

In order to simulate the regulatory impact, the pattern of node 8 is modelled as a linear combination of the patterns of node 1 and 2:

*X*_8t_ =*β*_1_*X*_1_*_t_*+*β*_2_*X*_2_*_t_*

where, *β*_1_ and *β*_2_ are the regulatory coefficients and follow independent standard normal distribution Normal (0,1^2^) in our experiments. Since node 8 was known to be regulated by node 1-5 according to the prior knowledge, it can be equivalently expressed as:

where, *β*_3_ = *β*_4_ = *β*_5_ = 0. Then, each expression level in a module is multiplied with a random number from Normal (0,1^2^ );this is used to simulate the difference in expression strength among genes. Finally, Gaussian additive noise was added and data were arranged in a matrix similar to microarray; rows of the data matrix were also shuffled to mimic real biological data.

The simulated system is similar to the one used in [14], except that additional network structure was specified. The goal of PATTERN is to reconstruct the embedded network structure and estimate the regulatory coefficients *β_i_* given the prior knowledge and gene expression data. In the following experiments, we evaluated the impacts of noise effect and prior knowledge on the performance of proposed PATTERN.

In the first experiments, PATTERN was evaluated under different noise variance by considering whether the correct network model was recovered and the percentage of correct model prediction is calculated. A network model is considered to be correctly identified when all the nodes within the network are predicted correctly, i.e., all the functional nodes are predicted to be functional, and all the nonfunctional nodes are predicted to be nonfunctional. For instance, in the network shown in Fig. 4, the network structure is correctly predicted if and only if nodes 1, 2, 8 are predicted functional, and meanwhile node 3-5 are predicted to be nonfunctional. (Node 6, 7 are independent nodes and thus are not considered.). The percentage of correct model prediction is defined as the ratio of the number of correct model predictions to the total number of experimental trials. For example, if among 100 trials, models are predicted correctly 80 times, the percentage of correct model prediction is 80%.

**Figure 5:**
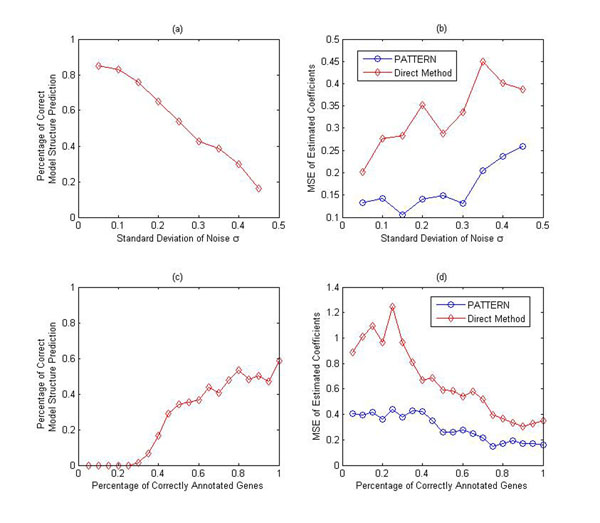
**PATTERN on small synthetic PathRNet** (a) The vertical axis represents the percentage of correctly predicted network structure and the horizontal axis denotes the noise standard deviation. The capability of PATTERN to recover the correct network structure decreases as noise increases. (b) The plot of the MSE of estimated coefficients vs. the noise standard deviation. PATTERN outperforms the direct method under all tested noise conditions. (c) Plot of the percentage of correctly predicted network structure vs. the percentage of correct annotation is a module. (d) Plot of the the MSE of estimated coefficients vs. the percentage of correct annotation is a module. (c) and (d) suggest that the ability of PATTERN to identify the correct network structure and estimate the coefficients was not significantly affected as long as more than 50% of coregulated genes are correctly annotated.

The result of first experiment is shown in Fig. 5-(a). It can be seen that PATTERN was able to identify the network structure correctly when noise is small, and the performance decreases as noise increases. To further evaluate the ability of PATTERN to estimate the regulatory coefficients *β_i_,* the mean squared error of estimated *β_i_* was calculated and compared with that of a direct method. The direct approach result is computed by considering the complete model, i.e., all the nodes are functional. In the network shown in Fig. 4, when using direct method, all five parent nodes 1-5 of node 8 in the network are considered, and the actual regression model is: *y = x*_1_*β*_1_*+ x*_2_*β*_2_ + *x*_3_*β*_3_ + *x*_4_*β*_4_ + *x*_5_*β*_5_ + *ε*. The direct method mimics the common practice of the Bayesian Networks based reconstruction approach and infers the regulatory relationship without considering node enrichment, thus involving all the nodes in inference. The comparison is shown in Fig. 5-(b). Compared with the direct method, PATTERN does improve the estimation accuracy of *β_i_* greatly under all tested noise conditions, which demonstrates the capability of PATTERN to identify the correct network structure and accurately estimate *β_i_.*

Note that in PATTERN the enrichment analysis serves as a model selection approach. The performance improvement of PATTERN over the direct method in *β_i_* estimation demonstrates the importance of model selection. As seen in this example, to recover the true network structure, we need to 1) remove the non-functional nodes 3-5 from network, and 2) decide whether node 1 and 2 regulates node 8. In PATTERN, objective 1) is achieved by the enrichment analysis in the context-specific PATTERN construction module, and objective 2) is tackled by the BIC model selection in regulatory PATTERN construction module. However, in the direct method, objective 1) is not tackled.

Then, we evaluated the impact of annotation database on performance. Even though great effort has been made to construct and improve various prior knowledge databases, the existing databases are still very noisy and often inconsistent with prior knowledge, i.e., annotation is often incomplete and contains error. This scenario was simulated by first assigning a function category only to a fraction of genes in an embedded module and leaving the rest without any annotation, and then introducing randomly selected genes into the function category. The validation result was shown in Fig. 5-(c) and (d). It can be seen from the figure that the ability of PATTERN to identify the correct network structure and estimate the coefficients was not significantly affected as long as more than 50% of coregulated genes are correctly annotated. PATTERN outperforms the direct method under all tested conditions. These results imply that PATTERN is robust to the incomplete and erroneous annotations in the prior knowledge databases. In this experiment, In order to maintain the network structure and acquire a comparable result, the annotations of genes that are shared in several pathways (and thus determine the edges and edge directions between nodes) are unchanged; only the annotation of remaining genes can change.

**Figure 6:**
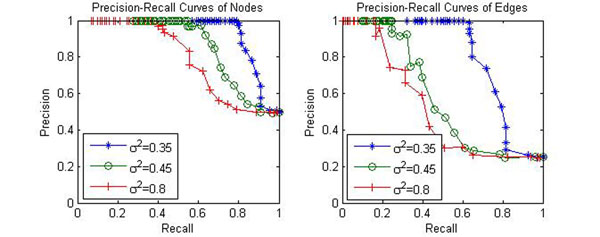
**Precision-Recall curves of (a) nodes and (b) edges of PATTERN on large synthetic PathRNet.** PATTERN was applied to a large simulated dataset, which consists of 4000 genes and 14 time samples. The PathRNet embedded consists of 60 nodes (30 functional and 30 nonfunctional) and 175 random edges (connectivity is equal to 5%, which means on average each node is regulated by 3 parent nodes).

Additional experiments were been conducted to test the performance of the proposed algorithm on more complicated network. The proposed algorithm was applied to a large dataset with 4000 genes 14 samples where a synthetic network with 60 nodes (30 functional nodes and 30 nonfunctional nodes) and around 175 random edges (0.05 connectivity) was embedded, and the regulatory coefficients follow independent standard normal distribution. This size of this network mimics a rather realistic situation. The precision-recall curves of reconstruction are plotted in Fig. 6.

It can be seen from the figure that, the proposed algorithm PATTERN achieves relatively better precision and recall performance at low noise level; its performance decreases as noise variance increases. Particularly, for the noise variance at 0.35, the precisions of nodes and edges drop below 1 only when respective recalls are above 0.8 and 0.65.

**Figure 7:**
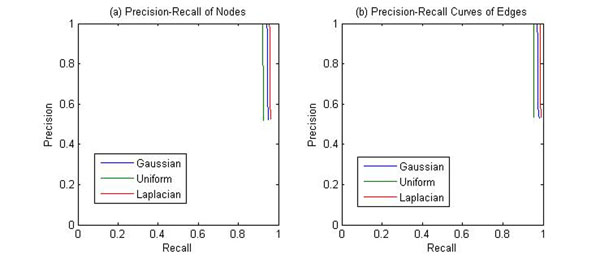
**Precision-Recall performance of (a) nodes and (b) edges of Pattern for the different noise distributions (small noise variance).** PATTERN was applied to a simulated dataset which consists of 400 genes 10 samples. The PathRNet embedded is shown in Fig. 4. Three kinds of noise distributions were added respectively, and the noise standard deviation is equal to 0.05.

**Figure 8:**
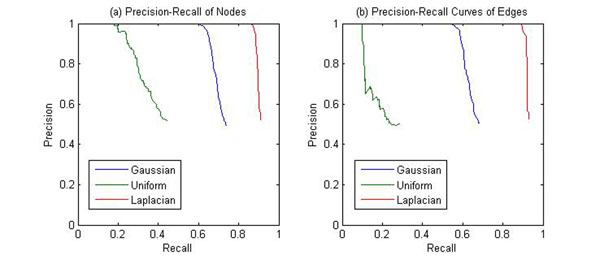
**Precision-Recall performance of (a) nodes and (b) edges of Pattern for the different noise distributions (large noise variance).** PATTERN was applied to a simulated dataset which consists of 400 genes 10 samples. The PathRNet embedded is shown in Fig. 4. Three kinds of noise distributions were added respectively, and the noise standard deviation is equal to 0.4.

To evaluate the proposed algorithm on different types of noise distributions, three different noise distributions (Gaussian, Laplacian and Uniform) are compared, and the results are shown in Figs 7 and 8.

It can be seen from the figures that, when noise variance is small, the proposed algorithm PATTERN performs similarly on the datasets for three different noise distributions; however, when noise variance is large, PATTERN performs much better on the Laplacian noise contaminated datasets than on the other two, which indicates that the proposed approach is more robust to heavy tail noise distributions when the standard deviation of noise is the same.

## Reconstruction of PathRNet for KSHV infection

We applied PATTERN to reconstruct a PathRNet for KSHV infection of human primary endothelial cells [19]. Previous studies have shown that KSHV infection of primary human umbilical vein endothelial cells (HUVEC) can be divided into two broad phases: entry phase and postentry phase. The entry phase is an acute event involving the binding of the virus glycoproteins to cellular receptors, delivery of virus encapsidated viral and cellular proteins and RNAs, and activation of various cellular pathways. The postentry phase is composed of a series of non-acute events involving the expression of viral genes and virus replication as well as subsequent virus packaging and egress (lytic) or establishment of viral latency, all of which closely interact with different cellular pathways and the expression of cellular genes. The process of KSHV infection including lytic replication and latency are known to be complicated with various cellular genes and pathways regulated during different time periods under different schemes. It is highly desirable and important to elucidate the dynamic regulations of pathways and gene expressions during infection. The detailed result of the reconstructed PathRNet for KSHV infection produced by PATTERN is discussed next.

### Generic PathRNet

**Figure 9:**
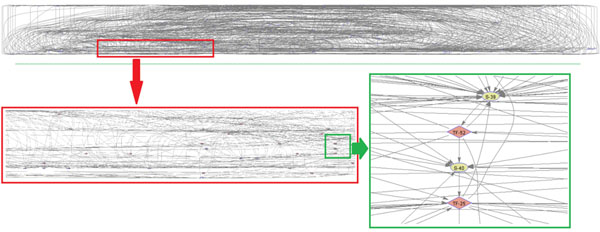
**Generic PathRNet** The generic PathRNet consists of 281 nodes and 1354 edges, which corresponds to 58 signaling pathways, 89 transcription factors, 134 metabolic pathways, and 1354 regulations. The generic PathRNet illustrates a global picture of the interactions between pathways and/or transcription factors in different human cells.

The generic PathRNet, as shown in Fig. 9, was constructed by integrating the existing knowledge derived from NCI-NC, Transfac and MSigDB [15-17], which includes 324 signaling pathways, 945 transcription factors, and 259 metabolic pathways. The constructed generic PathRNet consists of 281 nodes representing TFs, signaling / metabolic pathways and 1354 linkages, illustrating a generic picture of all the known interactions between pathways and transcription factors for different human cells under various conditions.

### Context-specific PathRNet revealed by ECTDISA

**Figure 10:**
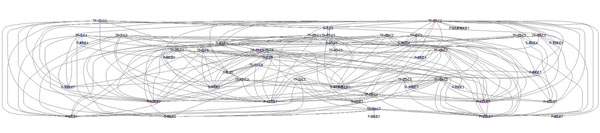
**Context-specific PathRNet** Context-specific PathRNet consists of 38 nodes and 204 edges, which are 38 enriched or functional pathways or TFs and 204 possible regulations during the KSHV infection of HUVEC.

A time series microarray data of KSHV infection of HUVEC [19] was introduced and the context-specific PathRNet (Fig. 10) for KSHV infection was reconstructed. The raw data consist of a total of 22283 features (probe set IDs) sampled at time *t =* [0,1,3,6,10,16,24,36,54,78] (hour) after infection. An intensity filter (the intensity of a gene should be above 100 in at least 1 sample) and a variance filter (the inter-quartile range of log2-intensities should be at least 0.2) were then applied to select 7159 differentially expressed features, which are further merged into 5650 Entrez gene database UIDs by taking the maximum value of all corresponding probe set IDs.

ECTDISA[18] was then applied to the time series data to obtain the coregulated gene clusters that are most enriched by the nodes of the generic PathRNet and at the same time retrieve the timing of coregulation.

Starting from 193 initial gene sets, ECTDISA identified 119 clusters, which were further merged into 7 modules according to the scheme in [18], and the 7 identified modules are shown in Fig. 3, among which the 2^nd^ module is a temporal module, whose coregulation is defined from 3^rd^ points to the last points. The enriched or functional components are listed in Table 1.

**Table 1:** Examples of enriched pathways and TFs in ECTDISA

Name	Annotation	Module
S-7	P38 MAPK SIGNALING PATHWAY	C5

S-23	ERK1/ERK2 MAPK SIGNALING PATHWAY	C5

TF-50	NF-YA	C2

TF-7	C-JUN	C3

P-128	GENES INVOLVED IN BASAL CELL CARCINOMA	C2

P-82	GENES INVOLVED IN DEGRADATION OF GLYCAN STRUCTURES	C7

Many of the enriched pathways and TFs, such as S-7, S-23, and TF-7 have been shown highly related to KSHV infection [21,25]. Please refer to Appendix for a complete list of enriched pathways and TFs.

**Figure 11:**
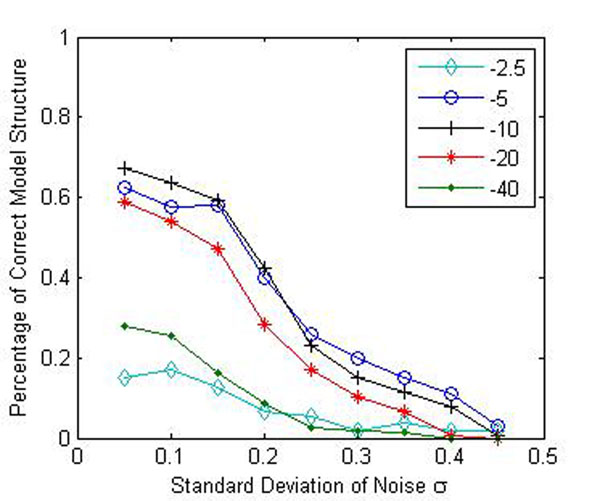
**Plot of noise standard deviation vs. sensitivity of reconstruction at different p-value thresholds.** PATTERN was applied to a simulated dataset which consists of 400 genes 10 samples. The PathRNet embedded is shown in Fig. 4. Different *p*-value thresholds are applied and compared.

Regarding how many nodes should be kept in the context-specific PathRNet, a node is considered to be significantly enriched in a module when the enrichment log p-value is larger than a predefined threshold. The threshold is chosen to be dependent on the noise level of the data. In general, a stricter threshold may result in too few nodes, i.e., low recall, and thus misses many true network connections; a relatively loose threshold may result in too many nodes, i.e., high false positive, and includes many false connections. As experiment was developed to show the sensitivity of the threshold and the result is shown in Fig. 11.

Specifically, PATTERN was applied to a simulated dataset which consists of 400 genes 10 samples. The PathRNet embedded is shown in Fig. 4. Different p-value thresholds are applied and compared. It can be seen from Fig. 11 that when the threshold is too strict (-40) or too loose (-2.5) the proposed algorithm cannot retrieve the true network structure. The result is better when a reasonable threshold is chosen. To this end, we used a p-value threshold of 0.01 to keep relatively more nodes in the network.

By keeping the only enriched or functional nodes (table 1) of the generic PathRNet, the context-specific PathRNet for KSHV infection was obtained. Since a number of enriched nodes are independent, those nodes are removed from the network. The reconstructed PathRNet consists of 38 nodes and 208 regulations (Fig. 10). Each node corresponds to a signaling pathway (S-), transcription factor (TF-) or a metabolic pathway (P-). The node enriched in the same cluster is highlighted by the same color and also indicated by the class ID "C#". Each directed edge represents the regulatory relationship.

### Regulatory and temporal PathRNet

The temporal PathRNets for the KSHV infection of HUVEC were constructed to reveal the dynamics in regulation at each sampling time of the microarray experiment.

**Figure 12:**
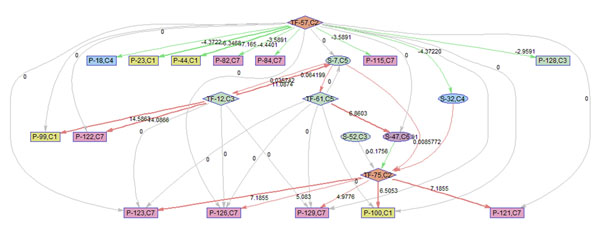
**Regulatory PathRNet** Only nodes that are close to P38 MAPK Signaling pathway or "S-7" are kept (distance<3). The color of the nodes indicates cluster attribute.

To better illustrate the result, a sub-network of related to p38 MAPK signaling pathway (S7) is acquired by keeping the nodes that are within the 2 nodes away from S-7. 9 frames of this sub-network are shown in Fig. 12. Please refer to Fig. 3 for the expression profiles of the involved clusters.

**Figure 13:**
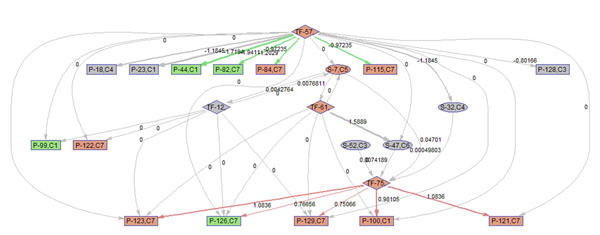
Temporal PathRNet (0 - 1 hpi)

**Figure 14:**
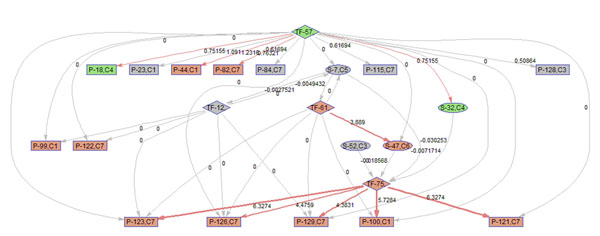
Temporal PathRNet (1 - 3 hpi)

**Figure 15:**
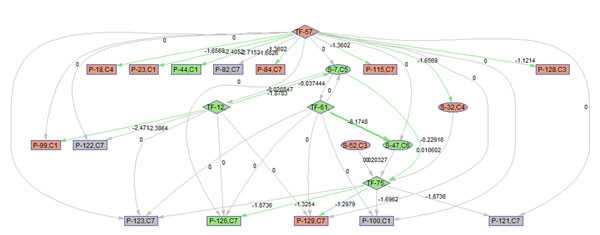
Temporal PathRNet (3 - 6 hpi)

**Figure 16:**
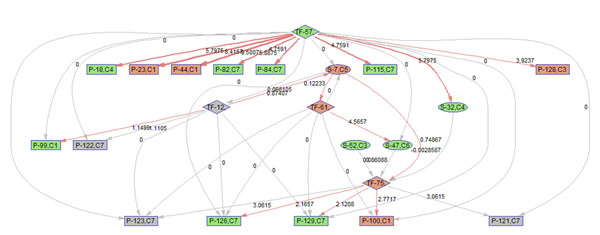
Temporal PathRNet (6 - 10 hpi)

**Figure 17:**
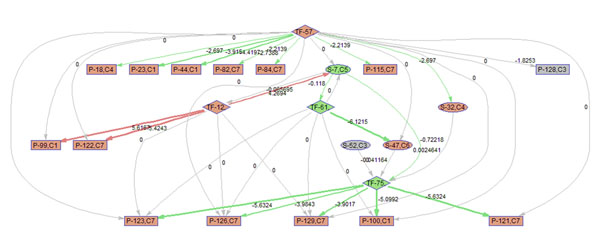
Temporal PathRNet (10 - 16 hpi)

**Figure 18:**
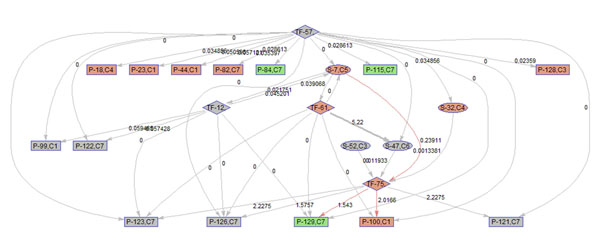
Temporal PathRNet (16 - 24 hpi)

**Figure 19:**
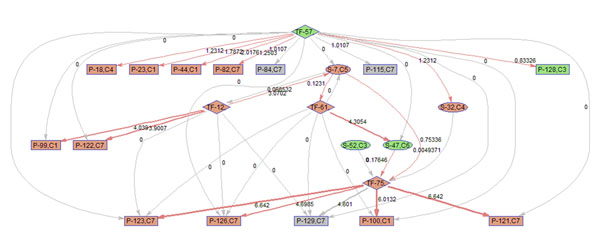
Temporal PathRNet (24 - 36 hpi)

**Figure 20:**
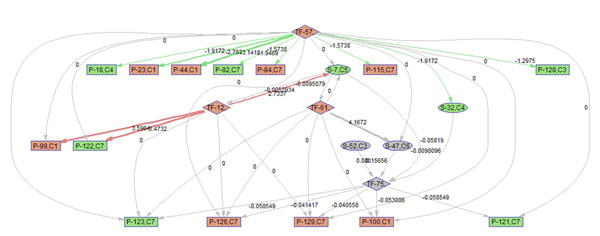
Temporal PathRNet (36 - 54 hpi)

**Figure 21:**
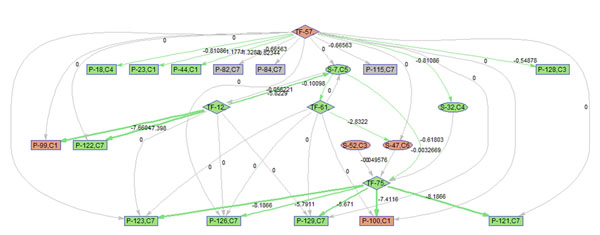
Temporal PathRNet (54 - 78 hpi)

First of all, the time varying nature of the network structure and regulatory impact are revealed in Fig. 13, 14, 15, 16, 17, 18, 19, 20, 21. The color of the nodes indicates the expression fold changes during the time period specified in Fig. 13, 14, 15, 16, 17, 18, 19, 20, 21 (Red represents up-regulation, and blue represents down-regulation). A directed linkage is determined by *β_p_* in Fig. 12, which is the regulation coefficient, and by  in Fig 13, 14, 15, 16, 17, 18, 19, 20, 21, which are the dynamic contributions of the parent nodes to the child node during the time period specified. (Red represents up-regulation, and green represents down-regulation, Please refer to Additional File 1, Appendix for the annotation of each node.)

In the Fig 13, 14, 15, 16, 17, 18, 19, 20, 21, the gray color of the nodes and links indicates that they are not functional. As shown by these figures, the colors of nodes and links vary over time, revealing the time-dependent characteristics of the network. Regarding the variation of the network structure, two aspects of the algorithm are important. First, the time-dependent module defines the start and the end of an enriched connection in the module. For instance, since P-128 is enriched in a temporal transcription module that is not defined between 0 to 3 h post infection (hpi), P-128 does not function in the first 2 frames of the time varying network (Fig. 13 and 14) and thus the nodes and links are in gray. However, they become active after 3 hpi (Fig. 15-16 and 20-21). Secondly, when the regulatory contribution of a parent node is sufficiently small, the parent node and the link are also marked gray to indicate nominal regulation. For example, while TF-75 regulates P-123, -126, -129 and -100 at different time points; it only regulates some or even none at some instances, say, between 3-6 hpi, or it only regulates P-129 and P-126 at other time points. Furthermore, it is easy to observe that the contributions for many nodes vary at different time points. Interestingly, a positive feedback loop exists between TF-12 and S-7.

Next, interesting regulatory cascades can be revealed by incorporating the annotations with the time varying networks. In Fig. 13 from 0-1hpi, the p38 MAPK pathway (S7) is up-regulated, which also induces the TF STAT1 (TF-61). This suggests that in the early stage of infection KSHV activates p38 MAPK pathway. Indeed, the activation of the p38 MAPK pathway is shown to be essential for KSHV infection and inhibition of the p38 MAPK pathway reduced KSHV infectivity [21]. Induction of STAT1 likely indicates the activation of cellular innate immune response to viral infection [22]. At 3 hpi in Fig. 14, it is suggested that STAT1 also activates SMAD2/3 signaling (S47), which is in agreement with the existing knowledge that activation MAPK pathway enhances Smad2 transcriptional activity [22]. In addition, TF CREB1 (TF-75) is also activated during both 1 and 3 hpi, which is expected since it is at the downstream of the MAPK pathway. Interestingly, the entire cascade from p38 MAPK to CREB1 becomes downregulated at 6 hpi. Following the first wave of activation of the cascade, we have also observed a second wave of activation of the p38 MAPK pathway and TF STAT1 at 10 hpi. The signal is again transduced to SMAD2/3 at 16 hpi accompanying the decay of the p38 MAPK pathway and TF STAT1, before being further transduced to CREB1 at 24 hpi. Activation of this second wave of the p38 MAPK pathway has indeed been described in [21,23].

## Discussion

PATTERN is a novel approach for analyzing large scale time series gene microarray datasets to uncover dynamic regulations of pathways and gene expression. We discuss next a few distinct features of PATTERN.

First, PATTERN relies on a robust and biological driven network architecture, PathRNet, which directly utilizes the existing functional databases. The enrichment scheme in PATTERN enables the identification of functional components of PathRNet, which enables the subsequent network inferences to focus on the context-specific part of the network structure, and thus significantly reduces inference complexity. As shown by the validation on the simulated network, PATTERN is also robust to the noisy and inconsistent annotations. All of these indicate the great capability of PATTERN to correctly identify the functional part of network structure.

Second, the time-varying network structure and dynamic temporal regulatory impacts can be inferred as a result of the ECTDISA, which not only identifies coregulated functional modules but also provides their timing information. This is critical information for all the related inference tasks in the reconstruction of dynamic network structure. Moreover, PATTERN can accurately estimate the dynamic regulatory weights.

Third, since the goal of PATTTERN is to uncover dynamic network structure, it is especially useful for analyzing non-stationary biological processes, where different regulations exist at different time periods. This is the motivation of studying KSHV infection. Compared with other biological processes such as cell cycles, temporal transcription patterns are more likely to exist in this dataset. In contrast, transcription patterns of cell cycle related genes are often expected to be cyclic and sinusoidal in shape, which also exist in the entire cell cycle process and are not time dependent by our definition.

Fourth, the computational complexity of the proposed PATTERN mainly lies in the context-specific PathRNet construction module, where biologically meaningful modules are retrieved by ECTDISA. The computational complexity of ECTDISA is proportional to the number of genes, the number of samples (conditions), the number of initial gene sets, the window length of the dependent analysis, the number of parameters to be optimized in the enrichment step, and the number of iterations required achieving convergence. (Please refer to [18,24] for the details)

## Limitations

Firstly, the performance of the proposed method partially relies on the quality of available prior knowledge. It cannot retrieve information of pathways that have not been annotated in existing knowledge databases. The absence of relatively complete and accurate prior knowledge may severely jeopardize its performance. However, as knowledge databases including GO, KEGG, and others continue to grow and improve, the proposed algorithm will improve its prediction performance accordingly.

Secondly, the proposed method cannot capture any intra-pathway interactions including feedback loops. More intelligent approaches are required to study the dynamics of intra-pathway associations. We will explore this modeling in our future research.

## Conclusions

A new paradigm that aims at reconstructing robust context-specific and time varying regulatory networks was proposed. The novelties of our approach that enable the discovery are: 1) A robust and biological driven network architecture, PathRNet, which builds upon the existing functional databases; 2) A functional enrichment based algorithm that assesses the enrichment and time span of the regulation of TFs and pathways in the context-specific time series microarray data. Due to PathRNet and the enrichment algorithm, we are able to robustly infer complex time-varying and nonlinear regulatory interactions that are otherwise impossible by the existing models and approaches. The resulting network provides unequivocal interpretation of biological functions and their impacts. The proposed approach for the first time provides a dynamic perspective of pathway, TF regulations, and their interactions related to specific endogenous and exogenous conditions. This context-specific, temporal landscape of regulation will help reveal directly the functional biomarkers that result in different phenotypes and consequently are important to drug design and therapeutic targets. In addition, this algorithm framework will enable the investigation of context specificity and time variation of other types of regulatory impacts such as miRNA regulation, thus capable of providing more complete knowledge of regulation.

## Competing interests

The authors declare that they have no competing interests.

## Authors’ contributions

JM, SJG, and YH conceived the idea. JM, YC, and YH worked out the detailed algorithms and derivations. JM implemented the algorithm and performed the testing. JM, ML, SJG, and YH wrote the paper.

## Supplementary Material

Additional File 1 Appendix I: Annotation of the nodes for Figure 5 Click here for file

## References

[B1] Deisboeck TS "Personalizing medicine: a systems biology perspective," Mol Syst Biol 2009 5 249 19293829 10.1038/msb.2009.8 PMC2671924

[B2] Van Someren EP Wessels LF Backer E Reinders MJ "Genetic network modeling," Pharmacogenomics 2002 vol. 3 pp. 507 25 10.1517/14622416.3.4.507 12164774

[B3] de Jong H "Modeling and simulation of genetic regulatory systems: a literature review," J Comput Biol 2002 vol. 9 pp. 67 103 10.1089/10665270252833208 11911796

[B4] Shmulevich I Dougherty ER Kim S Zhang W "Probabilistic Boolean Networks: a rule-based uncertainty model for gene regulatory networks," Bioinformatics 2002 vol. 18 pp. 261 74 10.1093/bioinformatics/18.2.261 11847074

[B5] Jansen R Yu H Greenbaum D Kluger Y Krogan NJ Chung S Emili A Snyder M Greenblatt JF Gerstein M "A Bayesian networks approach for predicting protein-protein interactions from genomic data," Science 2003 vol. 302 pp. 449 53 10.1126/science.1087361 14564010

[B6] Zhao W Serpedin E Dougherty ER "Inferring gene regulatory networks from time series data using the minimum description length principle," Bioinformatics 2006 22 2129 35 10.1093/bioinformatics/btl364 16845143

[B7] Rao A Hero Iii AO States DJ Engel JD "Inferring time-varying network topologies from gene expression data," EURASIP J Bioinform Syst Biol 2007 51947 18309363 10.1155/2007/51947 PMC3171343

[B8] Gupta M Qu P Ibrahim JG "A temporal hidden Markov regression model for the analysis of gene regulatory networks," Biostatistics 2007 vol. 8 pp. 805 20 10.1093/biostatistics/kxm007 17400597

[B9] Fujita A Sato JR Garay-Malpartida HM Morettin PA Sogayar MC Ferreira CE "Time-varying modeling of gene expression regulatory networks using the wavelet dynamic vector autoregressive method," Bioinformatics 2007 vol. 23 pp. 1623 30 10.1093/bioinformatics/btm151 17463021

[B10] Pal R Datta A Bittner ML Dougherty ER "Intervention in context-sensitive probabilistic Boolean networks," Bioinformatics 2005 vol. 21 pp. 1211 8 10.1093/bioinformatics/bti131 15531600

[B11] Luscombe NM Babu MM Yu H Snyder M Teichmann SA Gerstein M "Genomic analysis of regulatory network dynamics reveals large topological changes," Nature 2004 vol. 431 pp. 308 12 10.1038/nature02782 15372033

[B12] Myers CL Troyanskaya OG "Context-sensitive data integration and prediction of biological networks," Bioinformatics 2007 vol. 23 pp. 2322 30 10.1093/bioinformatics/btm332 17599939

[B13] Rachlin J Cohen DD Cantor C Kasif S "Biological context networks: a mosaic view of the interactome," Mol Syst Biol 2006 vol. 2 p. 66 10.1038/msb4100103 PMC1693461 17130868

[B14] Meng J Gao SJ Huang Y "Enrichment Constrained Time-Dependent Clustering Analysis for Finding Meaningful Temporal Transcription Modules," Bioinformatics 2009 10.1093/bioinformatics/btp235 PMC2687989 19351618

[B15] Subramanian A Tamayo P Mootha VK Mukherjee S Ebert BL Gillette MA Paulovich A Pomeroy SL Golub TR Lander ES Mesirov JP "Gene set enrichment analysis: a knowledge-based approach for interpreting genome-wide expression profiles," Proc Natl Acad Sci U S A 2005 vol. 102 pp. 15545 50 10.1073/pnas.0506580102 PMC1239896 16199517

[B16] Schaefer CF Anthony K Krupa S Buchoff J Day M Hannay T Buetow KH "PID: the Pathway Interaction Database," Nucleic Acids Res 2009 vol. 37 pp. D674 9 10.1093/nar/gkn653 PMC2686461 18832364

[B17] Wingender E "The TRANSFAC project as an example of framework technology that supports the analysis of genomic regulation," Brief Bioinform 2008 vol. 9 pp. 326 32 10.1093/bib/bbn016 18436575

[B18] Meng J Gao SJ Huang Y "Enrichment constrained time-dependent clustering analysis for finding meaningful temporal transcription modules," Bioinformatics 2009 vol. 25 pp. 1521 7 10.1093/bioinformatics/btp235 PMC2687989 19351618

[B19] Gao SJ Deng JH Zhou FC "Productive lytic replication of a recombinant Kaposi's sarcoma-associated herpesvirus in efficient primary infection of primary human endothelial cells," J Virol 2003 vol. 77 pp. 9738 49 10.1128/JVI.77.18.9738-9749.2003 PMC224610 12941882

[B20] Madeira SC Oliveira AL "Biclustering algorithms for biological data analysis: a survey," IEEE/ACM Trans Comput Biol Bioinform 2004 vol. 1 pp. 24 45 10.1109/TCBB.2004.2 17048406

[B21] Xie J Pan H Yoo S Gao SJ "Kaposi's sarcoma-associated herpesvirus induction of AP-1 and interleukin 6 during primary infection mediated by multiple mitogen-activated protein kinase pathways," J Virol 2005 vol. 79 pp. 15027 37 10.1128/JVI.79.24.15027-15037.2005 PMC1316010 16306573

[B22] Levy DE Garcia-Sastre A "The virus battles: IFN induction of the antiviral state and mechanisms of viral evasion," Cytokine Growth Factor Rev 2001 vol. 12 pp. 143 56 10.1016/S1359-6101(00)00027-7 11325598

[B23] Sadagopan S Sharma-Walia N Veettil MV Raghu H Sivakumar R Bottero V Chandran B "Kaposi's sarcoma-associated herpesvirus induces sustained NF-kappaB activation during de novo infection of primary human dermal microvascular endothelial cells that is essential for viral gene expression," J Virol 2007 vol. 81 pp. 3949 68 10.1128/JVI.02333-06 PMC1866142 17287275

[B24] Bergmann S Ihmels J Barkai N "Iterative signature algorithm for the analysis of large-scale gene expression data," Phys Rev E Stat Nonlin Soft Matter Phys 2003 vol. 67 p. 031902 10.1103/PhysRevE.67.031902 12689096

[B25] Ye FC Blackbourn DJ Mengel M Xie JP Qian LW Greene W Yeh IT Graham D Gao SJ "Kaposi's sarcoma-associated herpesvirus promotes angiogenesis by inducing angiopoietin-2 expression via AP-1 and Ets1," J Virol 2007 vol. 81 pp. 3980 91 10.1128/JVI.02089-06 PMC1866109 17287278

