# Supplementary material for: Robust inference of the context specific structure and temporal dynamics of gene regulatory network
Source: BMC Genomics. 2010 Dec 1;11(Suppl 3):S11. doi: 10.1186/1471-2164-11-S3-S11 (PMC2999341; doi:10.1186/1471-2164-11-S3-S11)
Supplement: Additional File 1 — Appendix I: Annotation of the nodes for Figure 5 [file 1471-2164-11-S3-S11-S1.doc]

# Appendix I: Annotation of the nodes for Figure 5

“S-” represent signaling pathway.

“TF-” represent transcription factor.

“P-” represent metabolic pathway.

S-1 > hypoxia and p53 in the cardiovascular system

S-6 > tumor suppressor arf inhibits ribosomal biogenesis

S-7 > p38 mapk signaling pathway

S-11 > cell cycle: g1/s check point

S-23 > erk1/erk2 mapk signaling pathway

S-32 > FOXA2 and FOXA3 transcription factor networks

S-39 > Calcineurin-regulated NFAT-dependent transcription in lymphocytes

S-40 > IL4-mediated signaling events

S-47 > Regulation of nuclear SMAD2/3 signaling

S-50 > Calcium signaling in the CD4+ TCR pathway

S-52 > HIF-1-alpha transcription factor network

S-53 > IL6-mediated signaling events

TF-1 > AP-2alphaA

TF-3 > C/EBPalpha

TF-6 > c-Fos

TF-7 > c-Jun

TF-9 > NF-Y

TF-10 > NF-YB

TF-12 > ATF-2-xbb4

TF-15 > Egr-1

TF-16 > ER-alpha

TF-22 > IRF-1

TF-24 > NF-A

TF-25 > C/EBPbeta

TF-26 > NF-kappaB

TF-27 > p50

TF-28 > RelA-p65

TF-31 > p53

TF-37 > USF1

TF-43 > STAT3

TF-50 > NF-YA

TF-57 > Sp3

TF-61 > STAT1

TF-68 > p53-isoform1

TF-75 > CREB1

P-18 > Genes involved in methionine metabolism

P-23 > Genes involved in arginine and proline metabolism

P-34 > Genes involved in starch and sucrose metabolism

P-44 > Genes involved in glycerolipid metabolism

P-72 > Genes involved in folate biosynthesis

P-82 > Genes involved in degradation of glycan structures

P-84 > Genes involved in neurodegenerative diseases

P-93 > Genes involved in regulation of autophagy

P-98 > Genes involved in gap junction

P-99 > Genes involved in complement and coagulation cascades

P-100 > Genes involved in antigen processing and presentation

P-115 > Genes involved in Huntingtons disease

P-121 > Genes involved in colorectal cancer

P-122 > Genes involved in renal cell carcinoma

P-123 > Genes involved in pancreatic cancer

P-126 > Genes involved in prostate cancer

P-128 > Genes involved in basal cell carcinoma

P-129 > Genes involved in melanoma
